# Liver cyst penetration of antibiotics at the target site of infection: a randomized pharmacokinetic trial

**DOI:** 10.1093/jac/dkae394

**Published:** 2024-11-07

**Authors:** Lucas H P Bernts, Roger J M Brüggemann, Anouk M E Jansen, Nynke G L Jager, Heiman F L Wertheim, Joost P H Drenth, Marten A Lantinga

**Affiliations:** Department of Gastroenterology and Hepatology, Research Institute for Medical Innovation, Radboud University Medical Center, Nijmegen, The Netherlands; Department of Medical Microbiology, Research Institute for Medical Innovation, Radboud University Medical Center, Nijmegen, The Netherlands; European Reference Network on Hepatological Diseases (ERN RARE-LIVER), Hamburg, Germany; Department of Pharmacy, Research Institute for Medical Innovation, Radboud University Medical Center, Nijmegen, The Netherlands; Department of Pharmacy, Research Institute for Medical Innovation, Radboud University Medical Center, Nijmegen, The Netherlands; Department of Pharmacy, Research Institute for Medical Innovation, Radboud University Medical Center, Nijmegen, The Netherlands; Department of Medical Microbiology, Research Institute for Medical Innovation, Radboud University Medical Center, Nijmegen, The Netherlands; Department of Gastroenterology and Hepatology, Research Institute for Medical Innovation, Radboud University Medical Center, Nijmegen, The Netherlands; European Reference Network on Hepatological Diseases (ERN RARE-LIVER), Hamburg, Germany; Department of Gastroenterology and Hepatology, Amsterdam UMC, University of Amsterdam, Amsterdam Gastroenterology Endocrinology Metabolism, Amsterdam, The Netherlands; Department of Gastroenterology and Hepatology, Research Institute for Medical Innovation, Radboud University Medical Center, Nijmegen, The Netherlands; European Reference Network on Hepatological Diseases (ERN RARE-LIVER), Hamburg, Germany; Department of Gastroenterology and Hepatology, Amsterdam UMC, University of Amsterdam, Amsterdam Gastroenterology Endocrinology Metabolism, Amsterdam, The Netherlands

## Abstract

**Background:**

The EASL cystic liver disease guideline states that drug penetration at the site of infection (liver cyst) is essential for successful treatment, but pharmacokinetic (PK) data on cyst penetration are limited.

**Objectives:**

This study aims to investigate tissue penetration of four antibiotics in non-infected liver cysts and explores influencing factors.

**Methods:**

We performed a prospective, randomized single-dose PK-study. Before percutaneous drainage of a non-infected liver cyst, an intravenous (IV) dose of either ciprofloxacin and piperacillin/tazobactam (group 1); or co-trimoxazole (trimethoprim/sulfamethoxazole) and doxycycline (group 2) was given. Cyst fluid was collected during drainage. Blood samples were obtained before, during and after drainage (within 12 h). Drug concentrations were measured with a validated LC-MS/MS. Primary outcome was liver cyst penetration, defined as the cyst-fluid-to-plasma concentration ratio (%) expressed as median (IQR).

**Results:**

We included 20 patients, and 21 liver cysts were drained (group 1: *n* = 11, group 2: *n* = 10). Median drained cyst volume was 700 mL. Median time between infusion and drainage was 139 min (IQR 120–188 min). Median cyst-fluid-to-plasma concentration ratio was 4.2% (IQR 1.6%–8.9%) for ciprofloxacin, 0.3% (IQR 0.0%–1.3%) for piperacillin, 0.2% (IQR 0.0%–1.3%) for tazobactam, 12.2% (IQR 6.3%–16.1%) for trimethoprim, 0.4% (IQR 0.2%–3.8%) for sulfamethoxazole and 1.6% (IQR 0.9%–2.3%) for doxycycline. Time between trimethoprim infusion and cyst drainage was correlated with increased cyst-fluid-to-plasma concentration ratio (*P* < 0.01).

**Conclusions:**

Trimethoprim and ciprofloxacin have the highest penetration ratios amongst antibiotics tested. We found that liver cyst penetration varies widely between drugs after a single IV dose.

**Clinical trial number:**

NTR8499

The trial was originally registered in the Netherlands Trial Register (ID: NL7290), which was converted to the International Clinical Trials Registry Platform in 2022.

## Introduction

Bacterial infection of liver cysts is a severe complication of polycystic liver disease, and is especially prevalent in patients on immunosuppressants or somatostatin-analogues.^1^ Liver cyst infections require several weeks of antibiotic treatment, but failure of antibiotic treatment necessitates escalation of care in 48% of cases.^2^ In some patients, recurrent cyst infections even necessitate liver transplantation.^3^ The recently published practice guideline on cystic liver disease states that drug penetration is a key factor for treatment success.^4^

Liver cyst infections are primarily caused by Gram-negative bacteria, with *Escherichia coli* and *Klebsiella* species most often detected.^5,6^ Currently, third-generation intravenous (IV) cephalosporins or oral ciprofloxacin are first-line treatment.^4^ Unfortunately, additional strategies are necessary because of increasing antimicrobial resistance to first-line treatment or higher abundancy of intrinsically resistant pathogens (e.g. enterococci).^6,7^ This calls for alternative treatment strategies for patients with liver cyst infection, such as piperacillin/tazobactam, co-trimoxazole and doxycycline.

Successful treatment of bacterial infections depends on adequate antibiotic drug concentrations at the site of infection. Unfortunately, data on tissue penetration in liver cysts is limited and available data suggests a wide range in drug penetration. Ciprofloxacin appears to penetrate well in liver cyst fluid, but cefazolin and meropenem showed limited cyst penetration in previous studies.^8–11^

Our primary objective was to investigate the liver cyst penetration of antibiotics after administration of a single IV dose in patients without infection. We assessed drug levels of ciprofloxacin, piperacillin/tazobactam, trimethoprim/sulfamethoxazole and doxycycline in cyst fluid (obtained by percutaneous aspiration) and compared those with plasma concentrations. A secondary objective was to explore drug- and patient-related factors that influence liver cyst penetration.

## Methods

### Trial design

This prospective, randomized, pharmacokinetic (PK) trial was carried out at the Radboud University Medical Center, Nijmegen, the Netherlands. Participants were randomized into two groups in a 1:1 allocation ratio. Group 1 received ciprofloxacin and piperacillin/tazobactam, while group 2 received trimethoprim/sulfamethoxazole and doxycycline. There were no protocol amendments This study was reported according to the CONSORT reporting guidelines for randomized trials (Table S1, available as Supplementary data at *JAC* Online).^12^

### Study drug selection

The drug combinations per group were defined on the absence of PK drug–drug interactions at the level of phase I and phase II enzyme systems as well as transporter affinities. The lowest available dosages were used to limit potential adverse events. First, ciprofloxacin was chosen as a positive control because of expected liver cyst penetration.^8,9^ Piperacillin/tazobactam, only available as IV, was chosen for its broad Gram-negative spectrum and effect on *Enterococcus faecalis.*^13^ We selected trimethoprim/sulfamethoxazole for its Gram-negative spectrum and oral formulation, as an alternative for ciprofloxacin.^13^ Tetracyclines have a broad Gram-negative and -positive spectrum and share a similar molecular structure. Most tetracyclines in use require no dose adjustments for renal insufficiency and are well tolerated.^13^ Although not the first-choice tetracycline for Gram-negative bacteria, doxycycline was ultimately chosen for this study as it was readily available, and minocycline is only available as an oral formulation. In contrast to abscesses, liver cyst infections are very rarely caused by anaerobic bacteria, so clindamycin or metronidazole were deemed less relevant.^6,14^

### Participants

Adult patients who were diagnosed with symptomatic (e.g. pain, pressure sensations, early satiety) liver cyst(s), without current cyst infection and therefore had an indication for percutaneous aspiration sclerotherapy were suitable for inclusion. Exclusion criteria were: allergy or other contra-indications for study drug use; impossibility for an IV cannula and vena puncture; severe renal impairment with an estimated glomerular filtration rate (eGFR) < 30 mL/min/1.73 m^2^, use of antibiotics in the 7 days before aspiration sclerotherapy and any medical condition that may interfere with the conduct of the study in the opinion of the investigator. Patients were identified in the outpatient clinic by the treating physician. Written informed consent was obtained from all participants before inclusion. Patients were included between 2019 and 2020. Due to re-allocation of resources during the COVID-19 pandemic, analysis of collected samples was postponed and completed in 2023.

### Interventions

#### Procedures

Group 1 received single doses of IV ciprofloxacin (200 mg, in 30 min) followed by IV piperacillin/tazobactam (4000/500 mg, in 30 min). Group 2 received single doses of IV trimethoprim/sulfamethoxazole (160/800 mg, in 30 min) followed by IV doxycycline (200 mg, push-infusion in 5 min). Drugs were administered by a registered nurse. Time between drug administration and cyst aspiration was dependent on the schedule of the interventional radiologist and intervening emergency procedures, patients’ travel times to the hospital and availability of nurses. Patients were assigned to a provisional time window between infusion and drainage by the researcher to ensure variability for exploratory analyses.

Blood was sampled at three time-points (after drug infusion, simultaneous to cyst aspiration and 6–8 h after end of drug infusion). All samples were collected on ice and stored at 2–8°C up to 24 h before processing. Blood samples were centrifuged (5 min at 1900g), separating plasma from EDTA-blood. Plasma was stored at −40°C until analysis. Aspiration sclerotherapy was performed according to standard hospital protocol. Procedure details are described elsewhere.^15^ One or two liver cysts are aspirated per procedure, limited by the maximum amount of ethanol that can be used per intervention.^11,15^ All adverse events were monitored during hospital admission and at the follow-up appointment after 4–6 weeks.

### Measurements and PK-estimations

#### Baseline characteristics

Baseline characteristics included clinical characteristics (age, gender, body weight, body length, cyst location in the liver and estimated cyst volume determined with ultrasonography during screening). Blood parameters included haemoglobin, white blood cell count (WBC) (10^9^/L), creatinine (μmol/L) and Chronic Kidney Disease Epidemiology Collaboration eGFR (mL/min/1.73 m^2^) and total protein (g/L). Cyst fluid was analysed for erythrocyte count (10^9^/L), WBC count (10^9^/L), total protein (g/L) and pH.

#### Quantification of drug concentrations

Total plasma and cyst fluid concentrations were determined using LC-MS/MS (XEVO TQ-S, Waters, Etten-Leur, the Netherlands). The assays were validated according to the ‘Guideline on bioanalytical method validation’ provided by the European Medicines Agency, which is described in more detail in Supplementary File S1.^16^ We calculated the area-under-the-concentration-time curve extrapolated to infinity (AUC_0-inf_) and maximum plasma concentration (*C*_max_), by means of *post hoc* estimation using non-linear mixed effect modelling with NONMEM^®^ (version 7.5.1). The rationale for the chosen models is provided in Supplementary File S1.

### Outcomes

Primary outcome measure was liver cyst penetration, i.e. the concentration ratio of administered drugs at time of aspiration (second blood measurement). The ratio expresses the drug concentration in liver cyst fluid (mg/L) as a percentage of the plasma concentration (mg/L).

Secondary outcome measures were the ratio of cyst fluid concentration as a ratio of the estimated plasma AUC_0-inf_, and as a ratio of estimated plasma *C*_max_ (%).

Exploratory outcomes comprised the relationship between the primary outcome and (i) time between infusion and cyst drainage, (ii) left or right liver lobe, (iii) aspirated cyst volume, (iv) cyst fluid protein content, (v) cyst fluid erythrocyte count and (vi) cyst fluid pH.

Adverse events were queried by the treating physician during hospital stay and at the first follow-up visit, and scored according to the Common Terminology Criteria for Adverse Events.^17^

As a *post hoc* analysis, the antibiotic concentrations in liver cyst fluid were compared to different minimum inhibitory concentration (MIC) cut-offs: the clinical breakpoint, ECOFF and MIC50, provided by EUCAST or CLSI, for known liver cyst infection pathogens (i.e. *Escherichia coli*, *Klebsiella pneumoniae*, *Enterobacter cloacae* and *Enterococcus faecalis*).^18^

### Sample size

We planned to include 20 patients (10 per group). Owing to the explorative nature of this study, we did not perform a power calculation. The current sample size is in line with similar PK studies.^11,19^

### Randomization

Random allocation was performed by block-randomization without stratification, in a 1:1 allocation ratio, with block sizes of 4 and 2, resulting in a maximum of 10 patients per group. Allocation was performed with Castor (Castor EDC, Amsterdam, the Netherlands) by the researcher (L.B.). Allocation concealment or blinding was not deemed necessary for the primary outcome.

### Statistical methods

Descriptive variables are expressed as percentages (%) and number of patients (*n*), or median (IQR). Exploratory correlations were assessed by visual analysis of scatter plots and measured with univariate linear regression analysis, expressed as *R*-squared (*R*^2^). Medians were compared with the Mann–Whitney *U*-test. A *P* value <0.05 was considered statistically significant.

### Medical-ethical approval

The study was approved by the Medical Research Ethics Committee of the region Arnhem-Nijmegen (#2018-4683, approval date: 15 January 2019) and registered with EudraCT (#2018-003262-13). The trial was registered in the Netherlands Trial Register (ID: NL7290), which was converted to the International Clinical Trials Registry Platform (ID: NTR8499). All investigational products were ordered, prepared, labelled (according to GMP annex 13), stored and delivered by the Clinical Trials Unit of the department of Pharmacy, Radboud University Medical Center.

## Results

**Figure 1.**
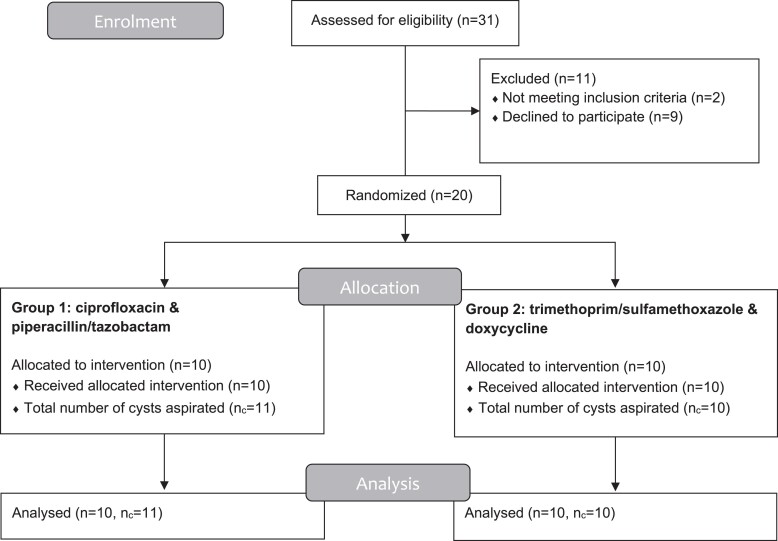
Flow diagram. Overview of enrolment, allocation and analysis of participants and number of aspirated cysts.

A total of 20/31 screened patients were included. One patient was excluded based on other medical conditions, one because of a history of difficulty with blood sampling and the other nine patients declined to participate (Figure 1). In one included patient, two cysts, of which one was relatively small (20 mL), were aspirated during the procedure. This resulted in a total of 21 liver cyst samples available for analysis. The median drained cyst volume was 700 mL (IQR: 375–1200 mL). Median time between infusion and drainage was 139 min (IQR 120–188 min; Figure S1).

Most patients were female (90%), had a solitary liver cyst (55%) and had an adequate renal function (95% with eGFR ≥60 mL/min/1.73 m^2^). Disease aetiology (70% versus 40% solitary cyst) and renal function (median >90 versus 80 mL/min/1.73 m^2^) were numerically different between groups; other baseline characteristics were overall comparable between groups (Table 1). Most aspirated liver cysts (62%) were located in the right hepatic lobe (Table 1).

**Table 1.** Baseline characteristics

Characteristic	Total group (*n* = 20; cysts = 21)	Group 1: ciprofloxacin and piperacillin/tazobactam (*n* = 10; cysts = 11)	Group 2: co-trimoxazole and doxycycline (*n *= 10; cysts = 10)
Age (years), median (IQR)	61 (55–68)	59 (44–65)	63 (55–72)
Female, *n* (%)	18 (90%)	9 (90%)	9 (90%)
Aetiology, *n* (%)			
Solitary cyst	11 (55%)	7 (70%)	4 (40%)
Polycystic liver			
ADPLD	7 (35%)	2 (20%)	5 (50%)
ADPKD	2 (10%)	1 (10%)	1 (10%)
Length (m), median (IQR)	1.69 (1.6–1.8)	1.71 (1.6–1.8)	1.66 (1.62–1.80)
Weight (kg), median (IQR)	70.9 (59.3–95.4)	74.0 (57.8–113.1)	66.4 (59.0–80.2)
BMI (kg/m^2^), median (IQR)	23.7 (20.7–30.0)	24.5 (20.1–35.7)	23.7 (21.8–27.7)
Renal function (eGFR), *n* (%)			
Normal (≥90)	9 (45%)	6 (60%)	3 (30%)
Mild (60–89)	10 (50%)	3 (30%)	7 (70%)
Moderate (30–59)	1 (5%)	1 (10%)	0 (0%)
eGFR (mL/min/1.73 m^2^), median (IQR)	88 (69 – >90)	>90 (81 – >90)	80 (64 – >90)
Cyst diameter (cm), median (IQR)	123 (91–145)	113 (98–134)	131 (87–174)
Aspirated cyst volume (mL), median (IQR)	700 (375–1200)	700 (470–800)	800 (288–2525)
Cyst fluid appearance, *n* (%)			
Clear	15 (71%)	8 (73%)	6 (67%)
Opaque	6 (29%)	3 (27%)	3 (33%)
Cyst location (segments), *n* (%)			
Right hepatic lobe (segment 5–8)	13 (62%)	6 (55%)	7 (70%)
Left hepatic lobe (segment 2–4)	8 (38%)	5 (45%)	3 (30%)
Caudate (1)	0 (0%)	0 (0%)	0 (0%)
Blood values, median (IQR)			
Haemoglobin (mmol/L)	8.3 (7.5–8.7)	8.5 (7.5–8.8)	8.0 (7.4–8.8)
White blood cell count (10^9^/L)	6.0 (5.0–7.0)	5.6 (4.3–7.4)	6.2 (5.4–6.7)
Total protein (g/L)	73.0 (71.0–76.0)	73.0 (71.5–75.5)	73.5 (70.8–77.3)
Creatinine (μmol/L)	68.0 (61.0–77.9)	66.0 (57.8–69.8)	76.5 (64.0–81.3)
eGFR (mL/min/1.73 m^2^)	87.5 (68.8 – ≥90)	≥90 (81 – ≥90)	80 (64 – ≥90)
Cyst fluid values, median (IQR)			
Erythrocytes (10^9^/L)	1.0 (0.0–9.0)	0.5 (0.0–11.0)	2.0 (0.0–54.5)
White blood cell count (10^9^/L)	0.0 (0.0–0.5)	0.1 (0.0–4.4)	0.0 (0.0–0.6)
Protein (g/L)	9.7 (5.0–22.6)	9.7 (4.8–22.0)	10.5 (5.0–28.7)
pH	7.6 (7.5–7.7)	7.7 (7.6–7.7)	7.6 (7.5–7.7)

Cyst diameter was measured with ultrasonography, the maximum diameter was reported. Large cysts can be located in multiple segments, there were no cysts in segment 1.

ADPLD, autosomal-dominant polycystic liver disease; ADPKD, autosomal-dominant polycystic kidney disease; BMI, body mass index; CKD, chronic kidney disease.

eGFR is based on the Chronic Kidney Disease Epidemiology Collaboration equation in mL/min/1.73 m^2^.

### Liver cyst penetration

**Figure 2.**
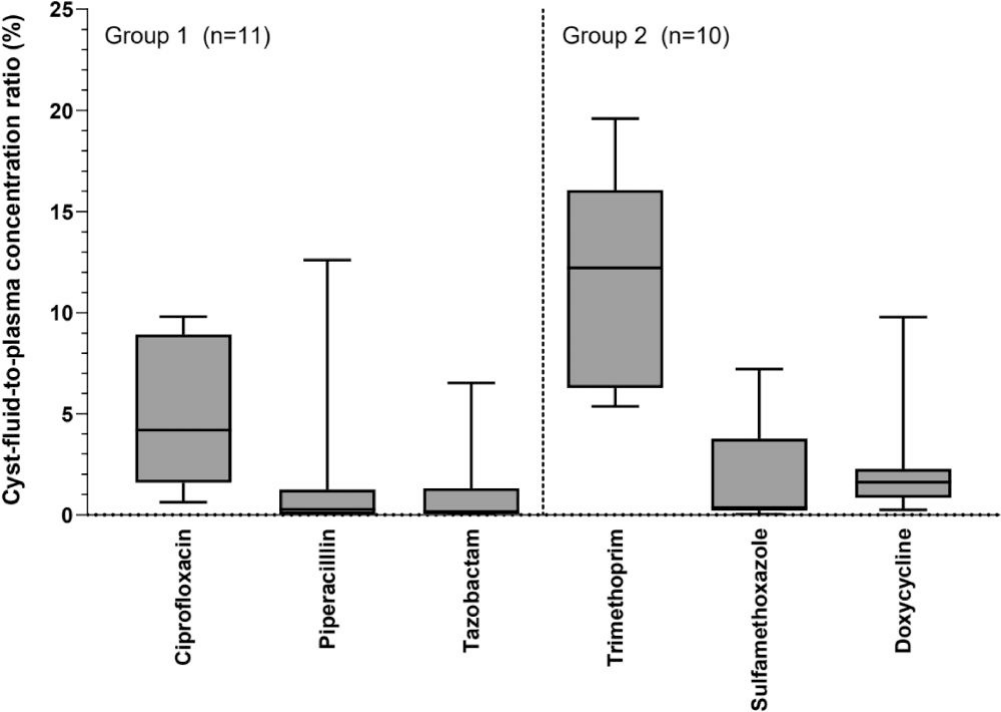
Cyst-fluid-to-plasma concentration ratio. On the *y*-axis is the cyst-fluid-to-plasma concentration ratio (%), presented as median, IQR (box) and total range (T-bars), for each study drug.

The primary outcome is shown in Figure 2, which illustrates the cyst-fluid-to-plasma concentration ratio for all drugs. Most cyst fluid measurements were below the plasma calibration curve (33 out of 61 cyst fluid samples). In group 1, for ciprofloxacin, median liver cyst concentration was 0.03 mg/L (IQR: 0.01–0.04 mg/L), which corresponds to a median cyst-fluid-to-plasma concentration ratio of 4.2% (IQR: 1.6%–8.9%). For piperacillin, median liver cyst fluid concentration was 0.05 mg/L (IQR: 0.02–0.91 mg/L), which corresponds to a median cyst-fluid-to-plasma concentration ratio of 0.3% (IQR: 0.0%–1.3%). For tazobactam, median liver cyst concentration was 0.01 mg/L (IQR: 0.00–0.10 mg/L), which corresponds to a median cyst-fluid-to-plasma concentration ratio of 0.2% (IQR 0.0%–1.3%).

In group 2, for trimethoprim, median liver cyst fluid concentration was 0.14 mg/L (IQR: 0.10–0.18), which corresponds to a median cyst-fluid-to-plasma concentration ratio of 12.2% (IQR 6.3%–16.1%). For sulfamethoxazole, median liver cyst fluid concentration of sulfamethoxazole was 0.14 mg/L (IQR: 0.09–1.19), which corresponds to a median cyst-fluid-to-plasma concentration ratio of 0.4% (IQR 0.2%–3.8%). For doxycycline, median liver cyst fluid concentration was 0.04 mg/L (IQR: 0.01–0.05), which corresponds to a median cyst-fluid-to-plasma concentration ratio of 1.6% (IQR: 0.9%–2.3%).

### Individual drug exposure

**Figure 3.**
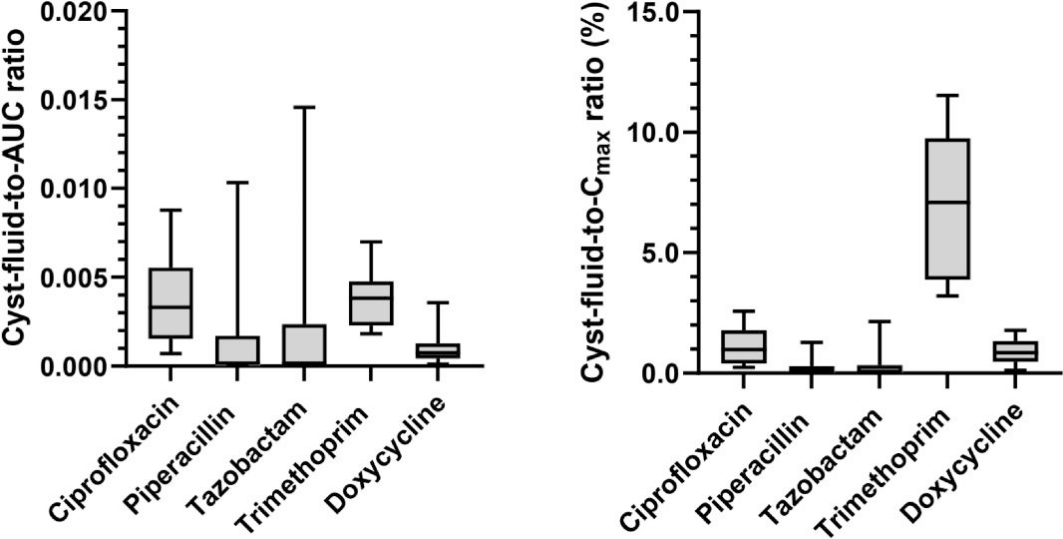
Cyst fluid concentration to estimated plasma AUC ratio, and cyst fluid concentration to estimated *C*_max_ ratio (%). Data are presented as median, IQR (box) and total range (T-bars). For ciprofloxacin, piperacillin, tazobactam: *n* = 11. For trimethoprim, doxycycline: *n* = 10.

Obtained plasma concentration-time curves are in line with known PK parameters for all drugs (Figure S1).^13^ The estimated individual drug exposure was consistent with known PK parameters for ciprofloxacin, piperacillin, tazobactam, trimethoprim and doxycycline (Table S2).^13^ Cyst fluid concentration to AUC_0-inf_ and to *C*_max_ (%) ratios showed comparable distributions to the primary outcome (cyst-fluid-to-plasma concentration ratio), with highest ratios for trimethoprim and ciprofloxacin (Figure 3).

### Exploratory analysis

**Figure 4.**
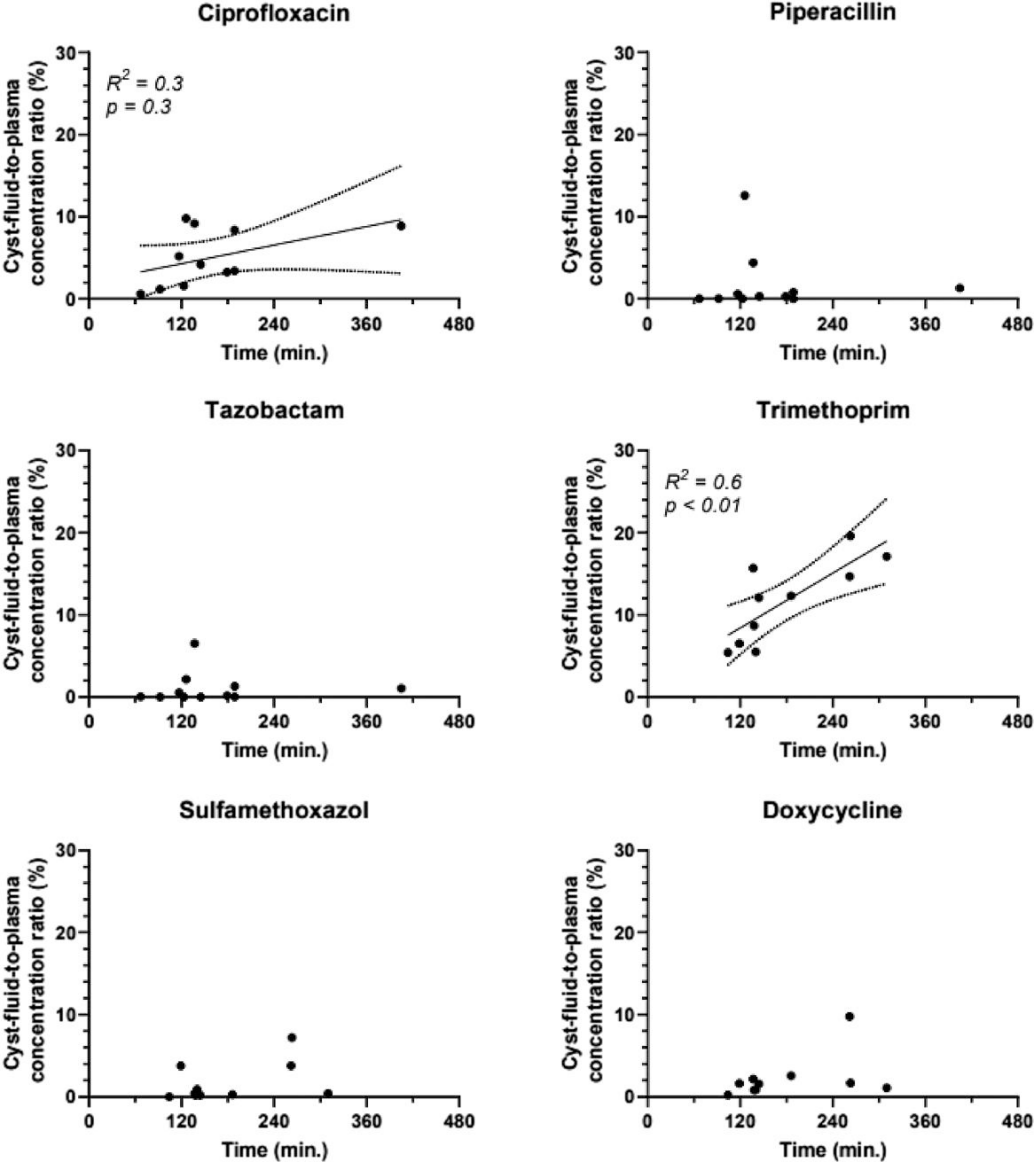
Liver cyst penetration over time. On the *y*-axes is the cyst-fluid-to-plasma concentration ratio (%). On the *x*-axes is the time between start of infusion and aspiration of cyst fluid in minutes. Interpolation line and 95% confidence interval, *R*^2^ and *P* value were calculated with linear regression for visual trends only.

Time between drug infusion and cyst drainage was correlated with an increased cyst-fluid-to-plasma concentration ratio for trimethoprim (*R*^2^ = 0.6; *P* < 0.01; Figure 4) and with increased trimethoprim intracystic concentrations (*R*^2^ = 0.7; *P* < 0.01). This correlation was not significant for ciprofloxacin (*R*^2 ^= 0.3; *P* = 0.1), piperacillin, tazobactam, sulfamethoxazole and doxycycline (Figure 4).

Median cyst-fluid-to-plasma concentration ratio was numerically higher in the left liver lobe than the right for all antibiotics except sulfamethoxazole and doxycycline, which was statistically significant for piperacillin (*P* = 0.03) and tazobactam (*P* = 0.02), but not for ciprofloxacin (*P* = 0.1) and trimethoprim (*P* = 0.3). Median time between infusion and cyst drainage was the same for cysts in the left or right lobe (141 versus 139 min.; *P* = 0.6).

There were no statistically significant correlations between aspirated volume and cyst-fluid-to-plasma concentration ratio. However, in the case where two cysts were aspirated, the concentration ratio was higher in a small, secondary cyst (20 mL) compared to the large cyst (600 mL) for ciprofloxacin (8.4% versus 3.4%), piperacillin (0.8% versus 0.0%) and tazobactam (1.3% versus 0.0%).

Protein content in cyst fluid did not correlate with cyst-fluid-to-plasma concentration ratio for any of the drugs (*P* > 0.1). Cyst fluid pH was within a tight range (Table 1), precluding correlation analysis.

### Comparison to minimum inhibitory concentrations

In Table 2, the concentrations found in cyst fluid are compared to the clinical breakpoint, ECOFF and MIC50 of known liver cyst infection pathogens. Most measurements were below the clinical breakpoint and/or ECOFF. Ciprofloxacin concentrations were above the MIC50 for included Enterobacterales in six to eight of the samples (55%–73%). Trimethoprim monotherapy concentrations were below the MIC50 for included Enterobacterales in all samples. When assuming the presence of a fixed sulfamethoxazole concentration, concentrations above the MIC50 were reached for trimethoprim/sulfamethoxazole in 70% of samples.

**Table 2.** Comparison to MIC cut-offs

	Clinical breakpoint		ECOFF		MIC50	
Ciprofloxacin (200 mg, half-dose)						
	Concentration (mg/L)	*N* >	Concentration (mg/L)	*N* >	Concentration (mg/L)	*N* >
*Escherichia coli*	0.25	0/11	0.06	1/11	0.016	8/11
*Enterobacter cloacae*	0.25	0/11	0.06	1/11	0.03	6/11
*Klebsiella pneumoniae*	0.25	0/11	0.125	0/11	0.03	6/11
*Enterococcus faecalis*	n/a	n/a	4	0/11	1	0/11
Piperacillin/tazobactam^a^						
*Escherichia coli*	8	0/11	8	0/11	2	1/11
*Enterobacter cloacae*	8	0/11	8	0/11	2	1/11
*Klebsiella pneumoniae*	8	0/11	8	0/11	2	1/11
*Enterococcus faecalis*	n/a	n/a	16	0/11	4	0/11
Trimethoprim						
*Escherichia coli*	4^b^	0/10	2	0/10	0.5	0/10
*Enterobacter cloacae*	4^b^	0/10	2	0/10	0.5	0/10
*Klebsiella pneumoniae*	4^b^	0/10	2	0/10	0.5	0/10
Trimethoprim/sulfamethoxazole^a^						
*Escherichia coli*	2	0/10	0.5	0/10	0.125	7/10
*Enterobacter cloacae*	2	0/10	0.5	0/10	0.125	7/10
*Klebsiella pneumoniae*	2	0/10	0.5	0/10	0.125	7/10
Doxycycline						
*Escherichia coli*	4^c^	0/10	8	0/10	2	0/10
*Enterobacter cloacae*	4^c^	0/10	8	0/10	2	0/10
*Klebsiella pneumoniae*	4^c^	0/10	4	0/10	2	0/10
*Enterococcus faecalis*	n/a	n/a	1	0/10	8	0/10

The number of samples above the mentioned cut-off is reported (*N* >). Clinical breakpoint: bacteria with an MIC higher than the clinical breakpoint are considered resistant; parameter independent of local acquired-resistance patterns. Epidemiological cut-off value (ECOFF): bacteria with an MIC below the ECOFF are considered wild-type organisms; parameter independent of local acquired-resistance patterns. MIC50: median MIC of all included isolates; parameter is dependent on local acquired-resistance patterns.

^a^Breakpoints and ECOFF are expressed as piperacillin and trimethoprim concentrations.

^b^For uncomplicated UTI only.

^c^CLSI breakpoint.

### Adverse events

There were no adverse events related to the administration of the investigational products. There were no severe adverse events during the study.

## Discussion

This study provides PK data on ciprofloxacin, piperacillin/tazobactam, trimethoprim/sulfamethoxazole and doxycycline penetration in liver cysts. We show that penetration of antibiotics into the liver cyst is highly variable between drugs. Overall, trimethoprim (12.2%) and ciprofloxacin (4.2%) penetrate best. We detected a relevant mismatch in penetration for the components of co-trimoxazole. The penetration ratio for trimethoprim (12.2%) was notably higher than sulfamethoxazole (0.4%). Exploratory analyses suggest that liver cyst penetration may be influenced by both liver cyst location and time between drug infusion and drug measurement.

### Liver cyst penetration

In line with previous data, we found that tissue penetration was best for ciprofloxacin, and cyst fluid concentrations were above the MIC50 in more than half of samples, even in a single, low-dose (200 mg) setting.^8,9^ In contrast to a favourable cyst penetration for trimethoprim, we detected only low intracystic levels of sulfamethoxazole. The consequence of this might be a higher likelihood of therapeutic failure as monotherapy trimethoprim is only used for the treatment of urinary tract infections, and addition of sulfamethoxazole (co-trimoxazole) is mandatory in other settings as the suspected causative pathogens are not susceptible to trimethoprim only.^13^ This was also reflected in our comparison of trimethoprim concentrations to MIC50 values, where sulfamethoxazole is necessary to reach values above the MIC50. These findings support the limited clinical preference for co-trimoxazole in the management of liver cyst infections.^2^ Similarly, co-trimoxazole fails to eradicate *Escherichia coli* from renal cysts, which has been attributed to the inability of sulfamethoxazole to enter renal cyst fluid.^20^ Doxycycline showed lower penetration while Gram-negative bacteria require relatively high drug levels to be effective.^21^ As such, ciprofloxacin remains the drug of choice for liver cyst infections if pathogens are susceptible to fluoroquinolones.

### Drug-specific properties

The differences in physicochemical properties and transporter affinities of drugs may have contributed to the variable degree of tissue penetration as seen in this study. The parameters lipophilicity, tissue distribution, protein binding and biliary excretion were examined for the purposes of this study.

The lipophilicity of drugs partly determines distribution in the body. A common measure of lipophilicity is the octanol-water partition coefficient (LogP).^22^ Drugs with high liver cyst penetration in this study were both lipophilic [rimethoprim (LogP 0.91)] and hydrophilic (ciprofloxacin (LogP −0.86), doxycycline (LogP −3.3)].^23^ As such, lipophilicity by itself fails to explain the observed differences in liver cyst penetration.

By contrast, tissue distribution, expressed as volume of distribution (L/kg), appears to be a predictor of liver cyst penetration. Drugs with the highest liver cyst penetration in this study also had a high volume of distribution (trimethoprim 1.6 L/kg, ciprofloxacin 2.5 L/kg, doxycycline 1.6 L/kg).^24–26^ Drugs with negligible liver cyst penetration had a relatively low volume of distribution (piperacillin 0.2 L/kg, tazobactam 0.2 L/kg and sulfamethoxazole 0.2 L/kg).^24,27^

When a drug is highly bound to proteins in the bloodstream, it is less available in its free form to diffuse into tissues. As a result, its effective concentration in that tissue might be limited, even if a drug has a high affinity for a particular tissue.^22,28^ It is important to note that the relationship between protein binding and tissue penetration is not absolute and can vary based on the specific characteristics of the drug, target tissues and the barriers present in the body. Our results do not show a clear correlation between liver cyst penetration and drug protein binding, while we evaluated antibiotics over a wide range of protein binding: moderate binding for ciprofloxacin, piperacillin, tazobactam and trimethoprim (20% to 40%), and higher binding for sulfamethoxazole and doxycycline (66% and 90%).^24–27^

### Accumulation over time

We demonstrated that liver cyst penetration of trimethoprim increased when the interval between drug infusion and cyst drainage was longer (hysteresis), which accounted for 60% of observed variance in penetration. This implies that the concentration of antibiotics within the cyst will increase even as the drug is being eliminated from plasma. This accumulation over time may be caused by factors such as tissue acidity, drug-ionization, molecular changes in lipid or water solubility or active transport to target sites.^22^ The current study design, however, did not allow to determine the exact change in accumulation ratio over time per patient.

Owing to accumulation over time, the single-dose setting of this study does not necessarily reflect the condition after repeated administrations and calculating concentration ratios after a single dose at a single time point does not provide complete insight into the clinical effectiveness of the detected drug concentrations. However, it is a standardized way to compare all included antibiotics in this setting. Future PK studies should consider this mechanism when designing drug dosing regimens and timing sample collection, ideally using several drug doses and tissue measurements.

### Strengths

The main strength of this study is that we used a controlled setting to perform a randomized study with a single IV dose. This prevents the biases in clinical PK studies caused by variation in oral bioavailability, dosing changes and treatment duration. We were able to include 20 patients with a high participation rate among screened patients. We organized a multidisciplinary network of hepatologists, interventional radiologists, pharmacists and clinical microbiologists within our hospital, which resulted in precise timing of sampling and high-standard measurements. We used population pharmacokinetic models to calculate exposure for all drugs but sulfamethoxazole, which allowed us to use a limited sampling design with only three blood samples per patient.

### Limitations

In this study, we were limited by the drainage at a single time point, which does not allow a comprehensive pharmacokinetic analysis with time after drug administration as the dependent variable. This was partly resolved by varying the time between infusion and aspiration in the study groups, although one should be careful of inferring within-patient effects by assessing between-patient differences. Although the sample size of our study is limited, the data could act as a physiologically based modelling exercise to make steady-state predictions. Half of measured cyst fluid concentrations were below the plasma concentration curve, which makes the calculation of antibiotics with low penetration less precise, but this variation will be much smaller than the difference with high-penetration antibiotics as ciprofloxacin and trimethoprim.

As we used the lowest available IV dose for the included study drugs and only one dose was given, our data will not completely reflect the tissue penetration after multiple oral doses and a comparison of cyst concentrations to MICs may underestimate the in vivo effects.

We only measured total concentrations of antibiotics and did not measure the unbound fraction of antibiotics, which is the pharmacologically active portion. This was done as the included study drugs, except doxycycline, have a low protein binding. EMA and FDA dictate in their guidelines on the investigation of drug interactions that investigating protein binding is of added value if the protein binding exceeds 80% and 90% relatively.^29^

Volume of the cyst was not correlated with tissue penetration in this study. We hypothesized that the lower surface-to-volume ratio of large cysts would impede penetration. Patients referred to aspiration sclerotherapy generally possess large dominant liver cysts.^30^ We examined penetration of drugs in cysts with a fairly limited range (IQR 375–1200 mL) and it is possible that a dataset derived from the full range of cyst sizes would identify a correlation.

The population PK models used to measure individual antibiotic exposure and *C*_max_ values will not fit our specific study population perfectly, so there is some variation in the estimated values that must be conidered. Nevertheless, this is a very innovative approach to estimate important PK parameters.

## Conclusions

In conclusion, we observed the highest penetration with ciprofloxacin and trimethoprim after a single IV dose. The limited penetration of sulfamethoxazole may contribute to failure of co-trimoxazole in clinical settings and we advocate continuing to use ciprofloxacin as first-line treatment or IV-oral step down. A clinical trial should assess whether there is a role for trimethoprim as oral prophylaxis in patients with frequently recurring (liver) cyst infections. Because of the lack of alternatives in case of quinolone resistance, future research should investigate liver cyst penetration of amoxicillin/ampicillin, clavulanate, cephalosporins and carbapenems.

## Supplementary Material

dkae394_Supplementary_Data

## Supplementary data

File S1, Figure S1 and Tables S1 and S2 are available as Supplementary data at *JAC* Online.
