# Supplementary material for: Liver cyst penetration of antibiotics at the target site of infection: a randomized pharmacokinetic trial
Source: J Antimicrob Chemother. 2024 Nov 7;80(1):182–91. doi: 10.1093/jac/dkae394 (PMC11695873; doi:10.1093/jac/dkae394)
Supplement: dkae394_Supplementary_Data [file dkae394_supplementary_data.docx]

**Table S1: Reporting checklist for randomised trial.**

Based on the CONSORT guidelines.

|  |  | Reporting Item | Page Number |
| --- | --- | --- | --- |
| **Title and Abstract** |  |  |  |
| Title | [#1a](https://www.goodreports.org/reporting-checklists/consort/info/#1a) | Identification as a randomized trial in the title. | 1 |
| Abstract | [#1b](https://www.goodreports.org/reporting-checklists/consort/info/#1b) | Structured summary of trial design, methods, results, and conclusions | 2 |
| **Introduction** |  |  |  |
| Background and objectives | [#2a](https://www.goodreports.org/reporting-checklists/consort/info/#2a) | Scientific background and explanation of rationale | 3 |
| Background and objectives | [#2b](https://www.goodreports.org/reporting-checklists/consort/info/#2b) | Specific objectives or hypothesis | 3 |
| **Methods** |  |  |  |
| Trial design | [#3a](https://www.goodreports.org/reporting-checklists/consort/info/#3a) | Description of trial design (such as parallel, factorial) including allocation ratio. | 4 |
| Trial design | [#3b](https://www.goodreports.org/reporting-checklists/consort/info/#3b) | Important changes to methods after trial commencement (such as eligibility criteria), with reasons | n/a |
| Participants | [#4a](https://www.goodreports.org/reporting-checklists/consort/info/#4a) | Eligibility criteria for participants | 5 |
| Participants | [#4b](https://www.goodreports.org/reporting-checklists/consort/info/#4b) | Settings and locations where the data were collected | 4 |
| Interventions | [#5](https://www.goodreports.org/reporting-checklists/consort/info/#5) | The experimental and control interventions for each group with sufficient details to allow replication, including how and when they were actually administered | 5, 6, S1 |
| Outcomes | [#6a](https://www.goodreports.org/reporting-checklists/consort/info/#6a) | Completely defined prespecified primary and secondary outcome measures, including how and when they were assessed | 6,7 |
| Outcomes | [#6b](https://www.goodreports.org/reporting-checklists/consort/info/#6b) | Any changes to trial outcomes after the trial commenced, with reasons | n/a |
| Sample size | [#7a](https://www.goodreports.org/reporting-checklists/consort/info/#7a) | How sample size was determined. | 7 |
| Sample size | [#7b](https://www.goodreports.org/reporting-checklists/consort/info/#7b) | When applicable, explanation of any interim analyses and stopping guidelines | n/a |
| Randomization - Sequence generation | [#8a](https://www.goodreports.org/reporting-checklists/consort/info/#8a) | Method used to generate the random allocation sequence. | 7 |
| Randomization - Sequence generation | [#8b](https://www.goodreports.org/reporting-checklists/consort/info/#8b) | Type of randomization; details of any restriction (such as blocking and block size) | 7 |
| Randomization - Allocation concealment mechanism | [#9](https://www.goodreports.org/reporting-checklists/consort/info/#9) | Mechanism used to implement the random allocation sequence (such as sequentially numbered containers), describing any steps taken to conceal the sequence until interventions were assigned | 7 |
| Randomization - Implementation | [#10](https://www.goodreports.org/reporting-checklists/consort/info/#10) | Who generated the allocation sequence, who enrolled participants, and who assigned participants to interventions | 7 |
| Blinding | [#11a](https://www.goodreports.org/reporting-checklists/consort/info/#11a) | If done, who was blinded after assignment to interventions (for example, participants, care providers, those assessing outcomes) and how. | n/a |
| Blinding | [#11b](https://www.goodreports.org/reporting-checklists/consort/info/#11b) | If relevant, description of the similarity of interventions | n/a |
| Statistical methods | [#12a](https://www.goodreports.org/reporting-checklists/consort/info/#12a) | Statistical methods used to compare groups for primary and secondary outcomes | 8 |
| Statistical methods | [#12b](https://www.goodreports.org/reporting-checklists/consort/info/#12b) | Methods for additional analyses, such as subgroup analyses and adjusted analyses | 8 |
| **Results** |  |  |  |
| Participant flow diagram (strongly recommended) | [#13a](https://www.goodreports.org/reporting-checklists/consort/info/#13a) | For each group, the numbers of participants who were randomly assigned, received intended treatment, and were analysed for the primary outcome | 8; Fig.1 |
| Participant flow | [#13b](https://www.goodreports.org/reporting-checklists/consort/info/#13b) | For each group, losses and exclusions after randomization, together with reason | 8; Fig.1 |
| Recruitment | [#14a](https://www.goodreports.org/reporting-checklists/consort/info/#14a) | Dates defining the periods of recruitment and follow-up | 5 |
| Recruitment | [#14b](https://www.goodreports.org/reporting-checklists/consort/info/#14b) | Why the trial ended or was stopped | n/a |
| Baseline data | [#15](https://www.goodreports.org/reporting-checklists/consort/info/#15) | A table showing baseline demographic and clinical characteristics for each group | 21 |
| Numbers analysed | [#16](https://www.goodreports.org/reporting-checklists/consort/info/#16) | For each group, number of participants (denominator) included in each analysis and whether the analysis was by original assigned groups | 8-10 |
| Outcomes and estimation | [#17a](https://www.goodreports.org/reporting-checklists/consort/info/#17a) | For each primary and secondary outcome, results for each group, and the estimated effect size and its precision (such as 95% confidence interval) | 8-10 |
| Outcomes and estimation | [#17b](https://www.goodreports.org/reporting-checklists/consort/info/#17b) | For binary outcomes, presentation of both absolute and relative effect sizes is recommended | 8-10 |
| Ancillary analyses | [#18](https://www.goodreports.org/reporting-checklists/consort/info/#18) | Results of any other analyses performed, including subgroup analyses and adjusted analyses, distinguishing pre-specified from exploratory | 8-10 |
| Harms | [#19](https://www.goodreports.org/reporting-checklists/consort/info/#19) | All important harms or unintended effects in each group (For specific guidance see CONSORT for harms) | 11 |
| **Discussion** |  |  |  |
| Limitations | [#20](https://www.goodreports.org/reporting-checklists/consort/info/#20) | Trial limitations, addressing sources of potential bias, imprecision, and, if relevant, multiplicity of analyses | 14-15 |
| Generalisability | [#21](https://www.goodreports.org/reporting-checklists/consort/info/#21) | Generalisability (external validity, applicability) of the trial findings | 11-13 |
| Interpretation | [#22](https://www.goodreports.org/reporting-checklists/consort/info/#22) | Interpretation consistent with results, balancing benefits and harms, and considering other relevant evidence | 11-15 |
| **Other information** |  |  |  |
| Registration | [#23](https://www.goodreports.org/reporting-checklists/consort/info/#23) | Registration number and name of trial registry | 1 |
| Protocol | [#24](https://www.goodreports.org/reporting-checklists/consort/info/#24) | Where the full trial protocol can be accessed, if available | 1 |
| Funding | [#25](https://www.goodreports.org/reporting-checklists/consort/info/#25) | Sources of funding and other support (such as supply of drugs), role of funders | 17 |

None The CONSORT checklist is distributed under the terms of the Creative Commons Attribution License CC-BY. This checklist can be completed online using <https://www.goodreports.org/>, a tool made by the [EQUATOR Network](https://www.equator-network.org) in collaboration with [Penelope.ai](https://www.penelope.ai)

| **Table S2. Individual exposure estimates** | | | | | |
| --- | --- | --- | --- | --- | --- |
| **Antibiotics** | **AUC_0-inf_ (mg*h/L)** | **C_max_ (mg/L)** | **Cyst fluid conc. to AUC_0-inf_ ratio (%)** | **Cyst fluid conc. to C_max_ ratio (%)** |  |
| Ciprofloxacin | 9.3  (6.3 – 12.6) | 2.0  (1.2 – 7.7). | 0.3%  (0.2% – 0.6%) | 1.0%  (0.4% – 1.8%) |  |
| Piperacillin | 365.9  (254.4 – 439.0) | 259.5  (184.0 – 302.4) | 0.0%  (0.0% – 0.2%) | 0.0%  (0.0% – 0.3%) |  |
| Tazobactam | 50.9  (39.3 – 60.3) | 32.5  (23.1 – 37.8) | 0.0%  (0.0% – 0.2%) | 0.0%  (0.0% – 0.3%) |  |
| Trimethoprim | 38.8  (38.0 – 41.0) | 2.1  (1.8 – 2.5) | 0.4%  (0.2% – 0.5%) | 7.1%  (3.9% – 9.8%) |  |
| Sulfamethoxazole | n/a | n/a | n/a | n/a |  |
| Doxycycline | 46.0  (34.0 – 81.0) | 4.9  (3.4 – 8.7) | 0.1%  (0.0% – 0.1%) | 0.9%  (0.5% – 1.3%) |  |

***Legend****: All values are median (IQR).* ***Abbreviations****: AUC_0-inf_ = estimated area-under-the-concentration-curve, extrapolated to infinity. C_max_ = estimated maximum concentration*. *N/a = model not available.*

**Supplementary File S1**

*Quantification of drug concentrations – methods of validation*

Concentration range, accuracy range, within-day precision, between-day precision, were measured for all analytes in both plasma and cyst fluid. Waste material (cyst fluid) from other patients who did not receive antibiotics prior to aspiration was used for the preparation of Quality Control samples. Samples spiked with known amounts of the various antibiotics were measured on a plasma calibration curve. Cyst fluid measurements with concentrations lower than the plasma calibration curve (ciprofloxacin <0.05 mg/l; piperacillin <0.20 mg/l; tazobactam <0.15 mg/l, trimethoprim <0.05 mg/l; sulfamethoxazole <1.0 mg/l; doxycycline <0.06 mg/l) were interpreted according to the measured area for the primary outcome. Stability of the analytes in cyst fluid after two days at room temperature and after three freeze-thaw cycles was confirmed.

*Individual drug exposure estimation*

We searched literature for available population pharmacokinetic models. For trimethoprim and doxycycline, only a single model was retrieved.^1, 2^ For ciprofloxacin and piperacillin-tazobactam, multiple models were available. The final model was selected at the discretion of the researcher based on similarity in patient populations and good transferability to NONMEM.^3, 4^ No model was available for sulfamethoxazole. All models were subsequently coded in NONMEM. We transformed all reported %CV to log normal variances using $\sqrt{{(e}^{\omega^{2}}-1)}.$

*References*

1. Hopkins AM, Wojciechowski J, Abuhelwa AY et al. Population Pharmacokinetic Model of Doxycycline Plasma Concentrations Using Pooled Study Data. *Antimicrob Agents Chemother* 2017; **61**.

2. Welte R, Beyer R, Hotter J et al. Pharmacokinetics of trimethoprim/sulfametrole in critically ill patients on continuous renal replacement therapy. *J Antimicrob Chemother* 2020; **75**: 1237-41.

3. Wallenburg E, Ter Heine R, Schouten JA et al. An Integral Pharmacokinetic Analysis of Piperacillin and Tazobactam in Plasma and Urine in Critically Ill Patients. *Clin Pharmacokinet* 2022; **61**: 907-18.

4. Gieling EM, Wallenburg E, Frenzel T et al. Higher Dosage of Ciprofloxacin Necessary in Critically Ill Patients: A New Dosing Algorithm Based on Renal Function and Pathogen Susceptibility. *Clin Pharmacol Ther* 2020; **108**: 770-4.

**Figure S1**

**Supplementary figure 1: Antibiotic plasma concentrations over time per antibiotic.** On the y-axis plasma concentrations in mg/L. On the x-axis time between start of infusion and sampling in minutes. Measurements in a single patient are connected by dashed lines. In two patients, the third sample was unavailable. Black squares are time points during aspiration, which are used to calculate the primary outcome (other samples represented as circles). Patients received multiple antibiotics by randomization (group 1 vs. group 2).
